# Heterogeneous phenotype of a Chinese Familial WHIM syndrome with CXCR4^V340fs^ gain-of-function mutation

**DOI:** 10.3389/fimmu.2024.1460990

**Published:** 2024-11-07

**Authors:** Yu Huang, Lu Li, Ran Chen, Lang Yu, Shunkai Zhao, Yanjun Jia, Ying Dou, Zhiyong Zhang, Yunfei An, Xuemei Tang, Xiaodong Zhao, Lina Zhou

**Affiliations:** ^1^ National Clinical Research Center for Child Health and Disorders, Ministry of Education Key Laboratory of Child Development and Disorders, Children’s Hospital of Chongqing Medical University, Chongqing, China; ^2^ Chongqing Key Laboratory of Child Rare Diseases in Infection and Immunity, Children’s Hospital of Chongqing Medical University, Chongqing, China; ^3^ Department of Hematology Oncology, Children’s Hospital of Chongqing Medical University, Chongqing, China; ^4^ Department of Biology, School of Arts and Sciences, Tufts University, Medford, MA, United States; ^5^ Department of Rheumatism and Immunology, Children’s Hospital of Chongqing Medical University, Chongqing, China

**Keywords:** CXCR4 variant, gain-of-function, inborn error of immunity, WHIM syndrome, heterogeneous phenotype

## Abstract

**Background:**

WHIM syndrome is a rare, autosomal dominant inborn error of immunity characterized by warts, hypogammaglobulinemia, infection, and myelokathexis. It is caused mainly by heterozygous mutations at the C-terminus of the C-X-C chemokine receptor type 4 (CXCR4) gene.

**Methods:**

We described the detailed clinical, genetic, immunological and treatment characteristic of four WHIM patients from a single Chinese family.

**Results:**

Here, we report four patients from a family carrying a variant of *CXCR4* (c.1016_1017dupCT), which introduces a frameshift at codon V340, resulting in an extension of 14 amino acids (p.V340L fs*27). We provide an in-depth analysis of their clinical, genetic, immunological and treatment characteristic, noting that these patients exhibited an atypical clinical phenotype when compared to reported CXCR4^R334X^ patients. Additionally, the frameshift variant CXCR4^V340fs^ led to impaired receptor downregulation in patients’ PBMCs, and in HEK293T cells transfected with the variant plasmids.

**Conclusions:**

Our study provided detailed clinical features of four CXCR4^V340fs^ WHIM patients from one Chinese family who presented atypical phenotype and enrich the spectrum of WHIM syndrome.

## Introduction

WHIM syndrome is classified as an inborn error of immunity (IEI) and is characterized by the presence of human papillomavirus (HPV)-related warts, hypogammaglobulinemia, recurrent infections, and myelokathexis. Myelokathexis is defined as peripheral neutropenia resulting from the abnormal retention of mature neutrophils in the bone marrow (1–3). Nearly all cases of WHIM syndrome are attributed to heterozygous autosomal dominant mutations in the C-X-C chemokine receptor type 4 (CXCR4) gene (4), CXCR4 is widely expressed across various cell types, including mature leukocyte subtypes, epithelial cells, endothelial cells, hematopoietic progenitors, stromal fibroblasts, and cancer cells (5, 6). CXCR4 interacts with its unique natural ligand, CXCL12. The CXCR4/CXCL12 axis is critical for the homing and egress of leukocytes from bone marrow, as well as for directing leukocytes trafficking to the spleen and lymph nodes and facilitating B cell development in the bone marrow (7, 8).

Together with the recently reported international cohort of 66 WHIM patients (9) (the P1-P4 in our study refers to the P37-P40 of the literature), fewer than two hundred cases of WHIM syndrome have been reported overall. Thirty-seven distinct *CXCR4* variants have been identified, which including eight nonsense variants, twenty-seven frameshift variants, and two missense variants. Notably, all variants, except one, are located in the cytoplasmic C-tail of the receptor (9–12). The most common and well-studied WHIM variant is CXCR4^R334X^, a nonsense variant that results in the deletion of the C-terminal 19 amino acids of the CXCR4 receptor. This variant leads to enhanced CXCL12-mediated receptor signaling, delayed desensitization, and impaired receptor internalization (13, 14).

In this study we present a Chinese family (designated P1–P4, corresponding to P37-P40 in a large WHIM syndrome cohort) with WHIM syndrome caused by a *CXCR4* C-terminal variant. Prior literature, in which we participated, included only brief descriptions of clinical features and CXCR4 internalization assays using constructing cell lines. The clinical phenotype of WHIM syndrome displays significant heterogeneity; only a minority of patients (approximately 20–30%) exhibit all four classic clinical features (9). Furthermore, neutrophil counts may normalize intermittently during infections, creating challenges and delays in diagnosis. Therefore, we present and compare the clinical information, laboratory features and treatment with previous reported hotspot variant patients in detail, finding out our V340L fs27 variant patients presented heterogeneous clinical manifestations and received less treatment. These detailed clinical and laboratory descriptions and comparisons may assist clinicians in better recognizing and diagnosing Whim syndrome, particularly in patients with atypical phenotypes.

## Materials and methods

### Patients

A total of five patients (including P5) from two families were enrolled. Peripheral blood samples were collected from all patients, their parents, and age-matched healthy controls (HCs). All data (including pictures) provided informed consent. The study was approved by the Ethics Committee of the Children’s Hospital of Chongqing Medical University (2021–138) and was conducted in accordance with the principles of the Declaration of Helsinki.

### Genetic analysis

Genomic DNA was extracted from peripheral blood samples and sent to MyGenostics (Beijing, China) for gene sequencing. All *CXCR4* variants are based on the reference sequence NM_003467.2. The variants in *CXCR4* were confirmed by Sanger sequencing using the following primers: forward, 5’-AGGCCCTAGCTTTCTTCCAC-3’; reverse, 5’- CATACAGCAACTAAGAACTTGGC-3’.

### Quantification of TRECs and KRECs

Quantitative real-time reverse transcription polymerase chain reaction (RT-qPCR) for detecting T cell receptor excision circles (TRECs) and kappa-deleting recombination excision circles (KRECs) was performed as described previously (15).

### Plasmid construction and transfection

The cDNA sequence encoding CXCR4^WT^ was purchased from Youbao Biotechnology (Changsha, China). Plasmids containing CXCR4^V340fs^ and CXCR4^R334X^ were generated by overlap PCR using CXCR4^WT^ as a template, subcloned into the 7.1-pCMV-3×Flag vector and confirmed by Sanger sequencing. HEK293T cells (2.5×10^5^cells/well) were plated overnight in 24 well plates and transiently transfected with 500 ng of wild-type (WT) or variant plasmids using Polyethylenimine Linear (PEI; APExBIO, USA). All subsequent experiments were performed 24 h later. HEK293T cells were also used as a tool cell-line in previous study as its CXCR4 expression levels were extremely low (20fmol/mg of membrane protein) and easier transfection (7).

### Transwell

Transfected HEK293T cells (1×10^5^ cells) were seeded into the upper chambers of Transwell (8 μm pore size; Corning, USA). 10%FBS DMEM medium with 50 nM CXCL12 were added to the bottom chamber. After incubation for 6 h, cells were fixed with 4% formaldehyde at room temperature for 30 min, washed twice with PBS and stained with crystal violet for 20 min. Finally, cells in the upper chamber were wiped off with a cotton swab and photographed.

### Flow cytometry analysis

Expression of CXCR4 on the surface of PBMCs from patients and HCs were detected by flow cytometry using the following antibodies (purchased from BioLegend): anti-human FITC-CD3, Pacific Blue-CD4, BV510-CD8, PerCP-Cy5.5-CD19, APC-Cy7-Fixable Viability, PE-CXCR4 (clone: 12G5), and PE-mouse lgG2a κ (isotype control).

For the internalization assay, PBMCs or transfected HEK293T cells were cultured in 24 well plates containing RPMI 1640 or DMEM medium supplemented with 10% FBS, respectively. Cells were incubated in the presence or absence of 10 nM, 50 nM, 100 nM, 200 nM CXCL12 (PeproTech, USA) for 1 h, 2 h and 4 h at 37°C respectively, washed, resuspended, and stained with flow cytometry staining buffer. Expression of CXCR4 on the cell surface were detected by FACS Canto II cytometer, and the data were analyzed by FlowJo V10 software.

For the Phosflow-cytometry studies, PBMCs were stimulated with 50 nM CXCL12 for 0 or 5 min at 37°C. The cells were then fixed with 4% paraformaldehyde (Thermo, USA), permeabilized (Invitrogen, USA), and stained with p-AKT S473 (BD Biosciences, USA).

Peripheral blood lymphocyte subsets were assessed using the following antibodies: anti-human CD3, CD4, CD8, TCR αβ, TCR γδ, CD45RA, CD27, CXCR3, CCR6, CXCR5, CD19, CD24, CD38, IgD (all from BioLegend). The gating strategies for the T/B cell subpopulations, and the normal reference range, have been described previously (16).

### RT-PCR

RNA were extracted from peripheral blood of patients and HCs using Blood Total RNA miniprep kit (Axygen, USA) and reverse transcribed to cDNA. *CXCR4* mRNA levels were determined by Real-time Quantitative PCR (RT-PCR) using TB Green Premix ExTaq II (Takara, Japan). *CXCR4* primers were as follows: F: 5’-GGGCAATGGATTGGTCATCCT-3’, R: 5’-TGCAGCCTGTACTTGTCCG-3’.

### Human B cell Enzyme−Linked ImmunoSpot assay

Pertussis toxin (PT)-specific IgG and total IgG secreted by memory B cells were detected using a Human IgG ELISpotBASIC kit (BioLegend, USA). Briefly, PBMCs were pre-stimulated with the TLR7/8 agonist R848 (1 μg/mL) and recombinant human IL-2 (10 ng/mL), or with PBS only, for 5 days at 37°C/5% CO_2_ (17, 18). ELISpot plates were pre-coated overnight at 4°C with PT (0.1 mg/mL) and anti-human IgG (15 μg/mL). Cultured PBMCs were added to the plates at 37°C/5% CO_2_ overnight, and then removed. Biotinylated anti-IgG monoclonal antibodies, alkaline phosphatase, and BCIP/NBT-plus substrate were added to the wells for 2 h, 1 h, and 10 min, respectively. Finally, the plates were left to dry overnight in the dark prior to analysis by ImmunoSpot.

### Statistical analysis

Results are expressed as the mean ± SD. *P* values were calculated using an unpaired Student’s t-test. All statistical analyses were performed using GraphPad Prism 8. *P*<0.05 was considered significant.

## Results

### Clinical manifestations of the family with WHIM syndrome

**Figure 1:**
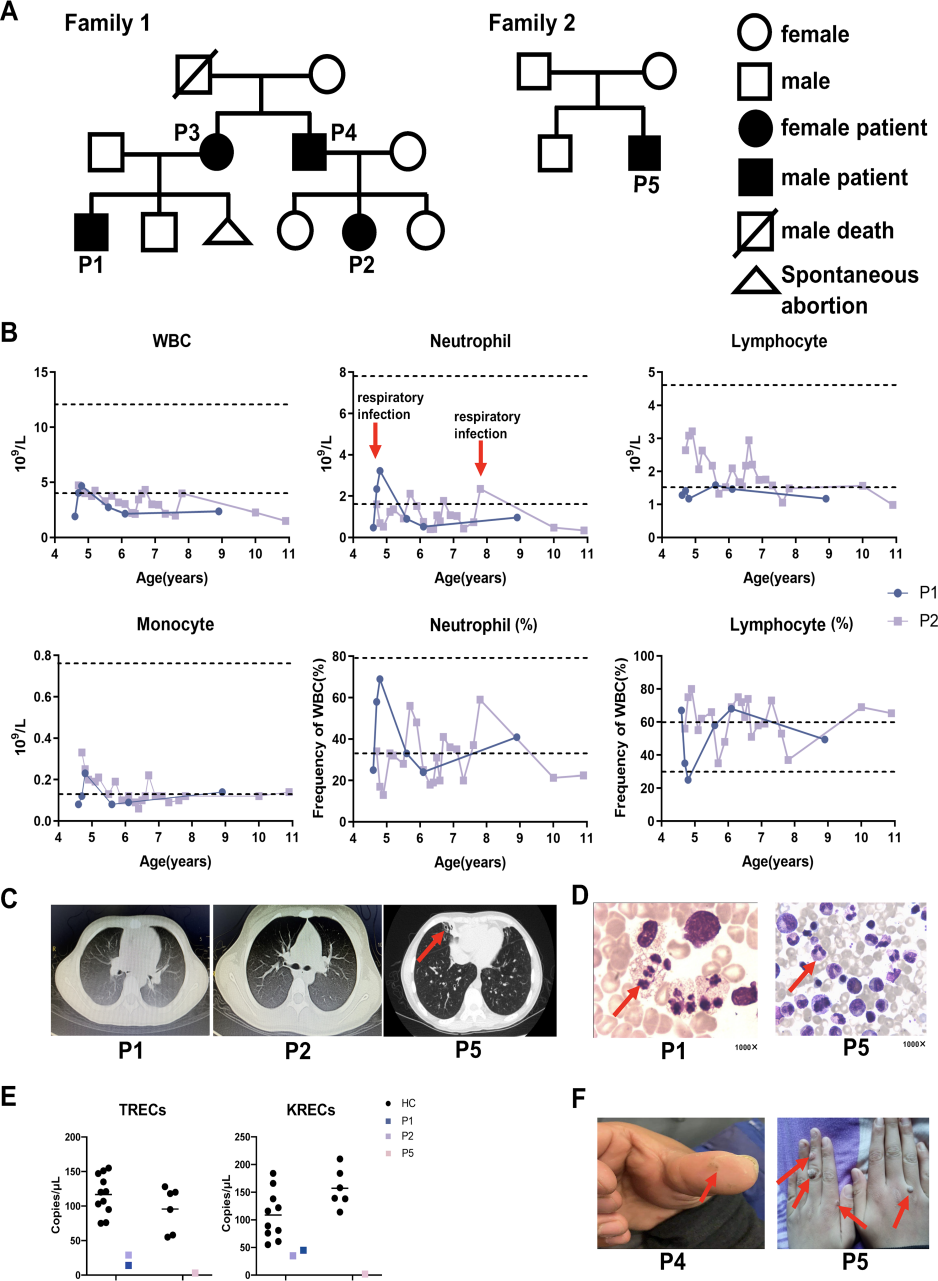
Pedigrees and clinical manifestations of 5 WHIM patients. **(A)** Pedigrees of two families with WHIM syndrome. **(B)** Hematological tests of P1 and P2. The dashed line represents the normal reference range. The red arrows represent respiratory infections. **(C)** The chest CT images of P1, P2 and P5. The red arrow indicates bronchiectasis of patient. **(F)** Skin warts on the hands of P4 and P5. The red arrow indicates warts of patients. **(D)** Bone marrow representative images of myelokathexis of P1 and P5 (1000**×**). The red arrow indicates myelokathexis. **(E)** The TRECS and KRECS of P1, P2 and P5 compared with age matched HCs.

A total of five patients were included in the study (**Figure 1A**). P1–P4 were members of the same family; notably, the father of P3 and P4 had passed away in his youth due to unknown causes. P5 was a patient from a different family (this case has been published previously (19) and served as a control for hotspot variants). All five patients presented with recurrent respiratory tract infections and neutropenia as their initial or primary symptoms.

P1 is currently 9.4 years old. He suffered from pneumonia at the age of 3 months, accompanied by neutropenia. Since that time, P1 has suffered respiratory infections approximately two to three times per year, primarily presenting with fever and cough, which typically resolve within a week after treatment with oral symptomatic medications or cephalosporin for infections. Between the ages of two and five, he experienced only two or three episodes of otitis media, with no associated hearing loss. Notably, he has never had an HPV infection. Hematological tests indicated a significant reduction in both neutrophils and lymphocytes; however, his neutrophil count transiently returned to normal during respiratory infections (**Figure 1B**). Immunoglobulin levels for P1 were within the reference range. Chest CT scans conducted at ages 4 years and 6 months and 10 years and 10 months revealed no abnormal findings (**Figure 1C**). Bone marrow examination demonstrated active granulocyte proliferation, with observed swelling, vacuolation, and toxic granules, consistent with myelokathexis (**Figure 1D**). Compared to age-matched healthy controls, P1 exhibited decreased TRECs and KRECs (**Figure 1E**).

P2 is currently 11.5 years old. She began exhibiting symptoms of respiratory tract infection, specifically recurrent fever and cough, at the age of 1 year. Since turning five, she had experienced only two or three episodes of otitis media, with no accompanying hearing loss. Notably, she has never had an HPV infection. When asymptomatic, a chest CT performed on P2 at the age of 10 years and 10 months revealed no abnormalities (**Figure 1C**). At the age of 4, P2 was hospitalized due to edema, and subsequent tests showed proteinuria, hyperlipidemia, and hypoproteinemia, leading to a diagnosis of nephrotic syndrome. Following treatment with oral prednisone, her urine protein levels quickly normalized. However, due to irregular follow-up and voluntary reduction of drugs, proteinuria recurred. She was then admitted to our hospital for further treatment. After regular follow-up and dosage adjustments over four years, routine urine examinations have shown no abnormalities. No renal manifestations were found in P1, P3 and P5.Neutropenia was detected when P2 was 4years old, and hematological tests revealed low leukocyte counts, particularly in neutrophils (**Figure 1B**). Immunoglobulin levels for P2 were within the reference range. Bone marrow tests were not performed for P2. Compared to age-matched healthy controls, both TRECs and KRECs were found to be decreased in P2 (**Figure 1E**).

P3 is currently 39 years old and has experienced recurrent cough and expectoration since childhood, with a diagnosis of neutropenia confirmed at the age of 24 years. P3 currently exhibits no symptoms of infection or skin warts. Her immunoglobulin levels fall within the reference range. Bone marrow examination in P3 reveals markedly active proliferation, characterized by a high proportion of mature granulocytes, which appear normal in size and morphology. These findings are consistent with myelokathexis.

P4, now 35 years old, has been prone to upper respiratory infections since the age of 2. He experienced otitis media two or three times, although neutropenia was not initially recorded. Subsequent hematological tests indicated leukopenia and neutropenia; however, these levels were not continuously monitored. A warty protuberance was observed on the pulp of P4’s left thumb (**Figure 1F**). Currently, P4 shows no symptoms of infection. No bone marrow examination or immunoglobulin level assessments have been conducted for P4.

P5 is currently 14 years old. He first experienced recurrent cough, expectoration, and intermittent fever at the age of 3, with neutropenia subsequently detected at 9 years old. He has suffered from recurrent otitis media and has been diagnosed with conductive deafness. A chest CT scan revealed bronchiectasis in the middle lobe of the right lung (**Figure 1C**). Additionally, multiple large verrucous vegetations were observed on the dorsum of P5’s hands (**Figure 1F**). His immunoglobulin levels are below the reference range. Bone marrow analysis showed active proliferation of nucleated cells in P5, with an increased granulocyte reserve characterized by a heightened myeloid-to-erythroid ratio and a “shift to the right.” The cells displayed signs of swelling, vacuolation, and the presence of toxic granules. These cytological changes in the bone marrow are indicative of myelokathexis (**Figure 1D**). Compared to age-matched healthy controls, both TRECs and KRECs were found to be decreased in P5 (**Figure 1E**). This finding aligns with previous reports of low TREC levels in certain WHIM patients (20, 21). Although all patients experienced recurrent infections, P1 - P4 did not develop bronchiectasis or hearing loss, suggesting that they may have had milder infections than P5; further studies involving more cases are warranted to explore this speculation. **Table 1** summarizes and compares the clinical characteristics of the five patients.

**Table 1:** Summary of the clinical and immunologic features of 5 patients.

	P1	P2	P3	P4	P5 (19)
Basic Information					
Age (years)	9.4	11.5	39	35	14
Sex	M	F	F	M	M
Age at onset (years)	0.3	1	childhood	2	3
Age at diagnosis (years)	4	7	33	31	11
Mutation	c.1016_1017dupCT	c.1016_1017dupCT	c.1016_1017dupCT	c.1016_1017dupCT	c.1000C>T
	p.V340Lfs*27	p.V340Lfs*27	p.V340Lfs*27	p.V340Lfs*27	p.R334X
Clinical manifestation					
Recurrent respiratory tract infections	+	+	+	+	+
Warts	–	–	–	+	+
Myelokathexis	+	ND	+	ND	+
Hypogammaglobulinemia	–	–	–	ND	+
Bronchiectasis	–	–	–	–	+
Recurrent otitis media	+	+	–	+	+
Hearing loss	–	–	–	–	+
Immunoglobulin					
IgG (g/L)	5.84 (5.28-21.9)	5.91 (5.28-21.9)	10.6 (7.51-15.6)	ND	**4.96↓** (5.28-21.9)
IgA (g/L)	0.71 (0.61-3.45)	0.636 (0.51-2.59)	2.33 (0.82-4.53)	ND	0.929 (0.61-3.45)
IgM (g/L)	0.973 (0.48-2.26)	1.69 (0.48-2.26)	1.64 (0.46-3.04)	ND	**0.186↓** (0.48-2.26)
IgE (IU/mL)	6.6 (<150)	41.6 (1-165)	ND	ND	2.6 (<150)

-, negative; +, positive; ND, not detected; The values in brackets are reference ranges. The values in bold indicate abnormal values. ↓ represent decreased.

Given the high heterogeneity observed in the clinical characteristics of WHIM patients, we reviewed the clinical manifestations, laboratory tests, treatments and outcomes of 28 hotspot mutation (R334X) patients and compared these items with the four patients (P1-P4) in our study. Detailed information is presented in **Table 2** and **Supplementary Table**, where positive clinical symptoms, laboratory test results, and treatments and outcomes are highlighted in gray. A comparative analysis reveals that the patients P1 - P4 from family1 only experienced respiratory tract infections and otitis media; notably, no infections involving the skin, gastrointestinal, urinary tract or other systems occurred. Additionally, there were no complications associated with recurrent severe infections, such as bronchiectasis and hearing loss, in patients P3 and P4 patients, in contrast to other mid-aged R334X patients). No congenital abnormalities or autoimmune diseases were found in patients P1-P4; in comparison, approximately 7% of patients with the R334X mutation reported autoimmune diseases, while around 14% exhibited congenital abnormalities. Moreover, no malignancies have been observed in P1-P4 patients to date. Laboratory tests indicated that neutropenia, lymphocytopenia and myelokathexis were found in nearly all patients. In terms of treatment and prognosis, our patients received general antibiotics solely for respiratory infections and did not undergo preventive anti-infective therapy. In contrast, 32% of patients with the hot R334X mutation were administered antibiotic prophylaxis. Furthermore, P1 received intravenous immunoglobulin (IVIG) treatment approximately 10 times from infancy to age 9, while P2 and P3 underwent IVIG treatment once and twice, respectively. P4 did not receive any IVIG treatment. In summary, the findings suggest that the four patients in our study exhibited relatively mild and heterogeneous clinical manifestations.

**Table 2:** Comparison of P5 with the hotspot mutation R334X with P1-P4.

		P1	P2	P3	P4	P5	R334X (n/N)
**Gender**		M	F	F	M	M	15F/8M
**Age at onset**		4m	1y	childhood	2y	3y	Born-29y
**Age at diagnosis**		4y	7y	33y	31y	11y	4m-52y
**Recurrent respiratory tract infections**		+	+	+	+	+	11/28
**Other infections**		–	–	–	–	–	13/28
**Infection-related sequelae**	Bronchiectasis	–	–	–	–	+	3/28
	Hearing loss	–	–	–	–	+	3/28
**Warts**		–	–	–	+	+	13/28
**Hypogammaglobulinemia**		–	–	–	ND	+	21/27
**Myelokathexis**		+	ND	+	ND	+	21/22
**Autoimmunity**		–	–	–	–	–	2/28
**Congenital abnormalities**		–	–	–	–	–	4/28
**Malignancy**		–	–	–	–	–	6/28
**Neutropenia**		+	+	+	+	+	28/28
**Lymphopenia**		+	+	+	+	+	17/28
**Treatment**	Antibiotic prophylaxis	–	–	–	–	+	8/25
	G-CSF	–	–	–	–	+	19/24
	R-IgGRT	–	–	–	–	–	9/24
	IR- IgGRT	+	+	+	–	+	3/24
	Plerixafor	–	–	–	–	–	7/24
	HSCT	–	–	–	–	–	1/24
**Outcome**	Asymptomatic	+	+	+	+	–	2/17
	Symptomatic survival	–	–	–	–	+	12/17
	Died	–	–	–	–	–	1/17
	Chromothrinsis	–	–	–	–	–	2/17

ND, not detected; R-IgGRT, regular IgG replacement treatment; IR- IgGRT, irregular IgG replacement treatment.

### Identification of a heterozygous extended variant in CXCR4

**Figure 2:**
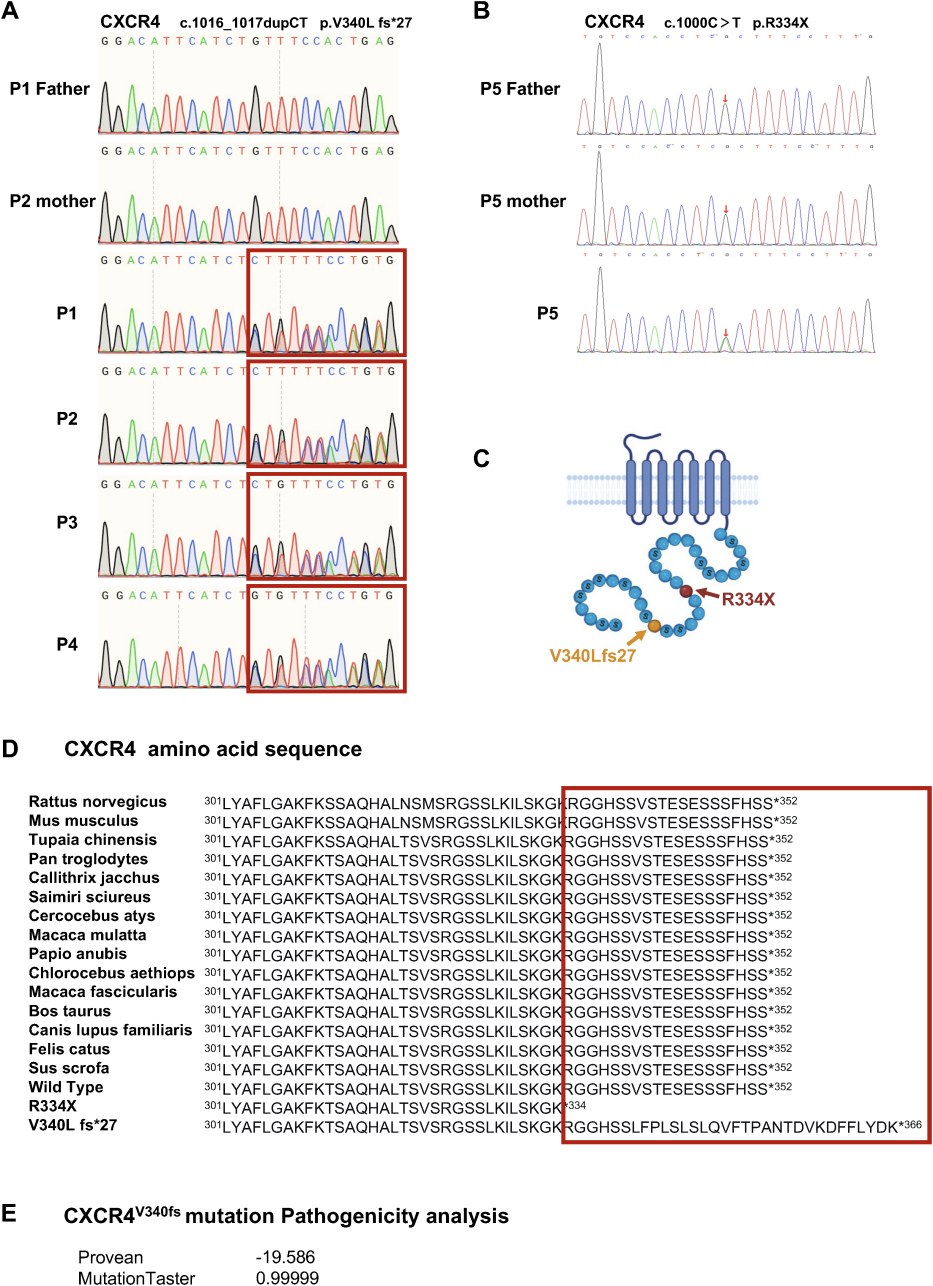
Detection of a heterozygous mutation of CXCR4 led to WHIM syndrome. **(A)** Sanger sequencing of *CXCR4* were performed using genomic DNAs from family 1. **(B)** Sanger sequencing of the CXCR4 gene in P5 and family members. **(C)** Schematic representation of seven transmembrane G protein coupled receptor CXCR4. Yellow, the variant of family 1. Red, the hotspot CXCR4 variant of WHIM syndrome. **(D)** The variant and WT amino acid sequences of CXCR4. The C-terminal of CXCR4 is highly conserved phylogenetically. **(E)** The pathogenicity analysis of the CXCR4^V340fs^ variant.

**I**mmunopanel analysis identified *CXCR4* variants in all five patients, which were subsequently confirmed by Sanger sequencing. P1–P4 from family 1 carried a heterozygous variant, c.1016_1017dupCT, in exon 2 of the CXCR4 gene. This variant results in a frameshift, leading to the protein alteration p.V340L fs*27. Following the guidelines established by the American College of Medical Genetics and Genomics (ACMG) for interpreting sequence variants, this variant was classified as pathogenic. P5 from family 2 harbored the most common WHIM-associated variant in the CXCR4 gene (c.1000C>T, p.R334X) (**Figures 2A, B**). Notably, these variant amino acids are situated within the highly conserved cytoplasmic C-terminal domain of the CXCR4 gene (**Figures 2C, D**). The mutation taster prediction indicated that CXCR4^V340fs^ was “pathogenic”, with a Provean prediction score of -19.586 (**Figure 2E**). Therefore, the clinical manifestations observed in the patients, combined with bioinformatics analysis, strongly suggest that the *CXCR4* (c.1016_1017dupCT, p.V340L fs*27) variant may be pathogenic.

### Decreased surface CXCR4 expression on CD8^+^T cells and B cells from patients

**Figure 3:**
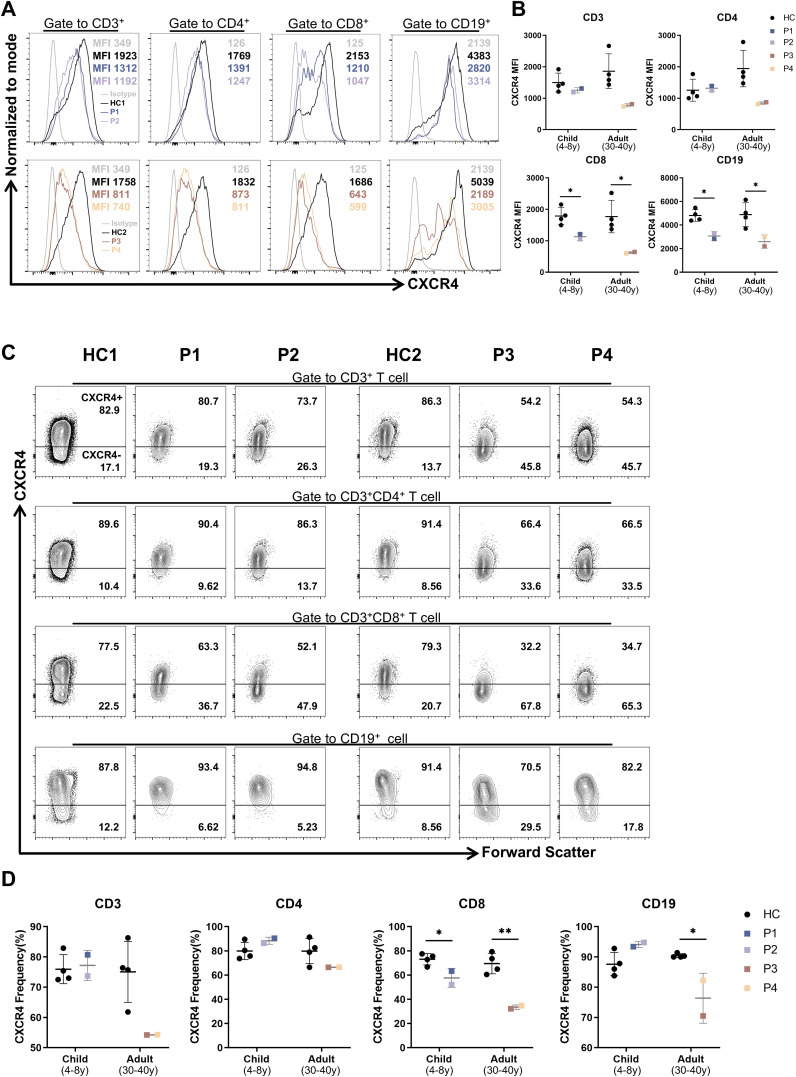
The surface CXCR4 expression were decreased on B cells and CD8^+^T cells in patients. **(A)** Mean fluorescence intensity (MFI) of CXCR4 on PBMCs from patients and age-matched HCs (n=4). **(B)** The CXCR4 MFI of lymphocyte subsets in panel **(A)**. **(C)** CXCR4^+^ proportion of PBMCs from patients and age-matched HCs (n=4). **(D)** Statistics of CXCR4^+^ proportion in panel **(C)**. *P<0.05, **P<0.01 for comparison between patients and HCs.

Given that CXCR4 is widely expressed on leukocytes (22) and previous studies have reported a decreased proportion of CXCR4^+^ cells along with lower CXCR4 mean fluorescence intensity (MFI) in PBMCs from WHIM patients (5), we investigated whether the CXCR4^V340fs^ heterozygous mutation affects receptor expression in PBMCs. We assessed the surface expression of CXCR4 using flow cytometry. For P1–P4, the MFI of CXCR4 on CD8^+^T cells and B cells was significantly lower compared to cells from age-matched HCs (**Figures 3A, B**). We also measured the transcript levels of *CXCR4* using RT-PCR. Interestingly, P1-P4 exhibited relatively higher levels of *CXCR4* expression, particularly in pediatric patients P1 and P2 (**Supplementary Figure SA**). Concurrently, the percentage of CXCR4^+^ cells within CD8^+^T cells significantly decreased, while the percentage of CXCR4^+^ cells among B cells in adult patients was lower than that in cells from HCs (**Figures 3C, D**). These findings indicate that the CXCR4^V340fs^ variant reduces the surface expression of CXCR4 on CD8^+^T cells and B cells. While these changes were a bit less visible in CD4+T and CD3+T cells.

### The CXCR4^V340fs^ variant results in impaired receptor internalization and hyperactive downstream signaling

**Figure 4:**
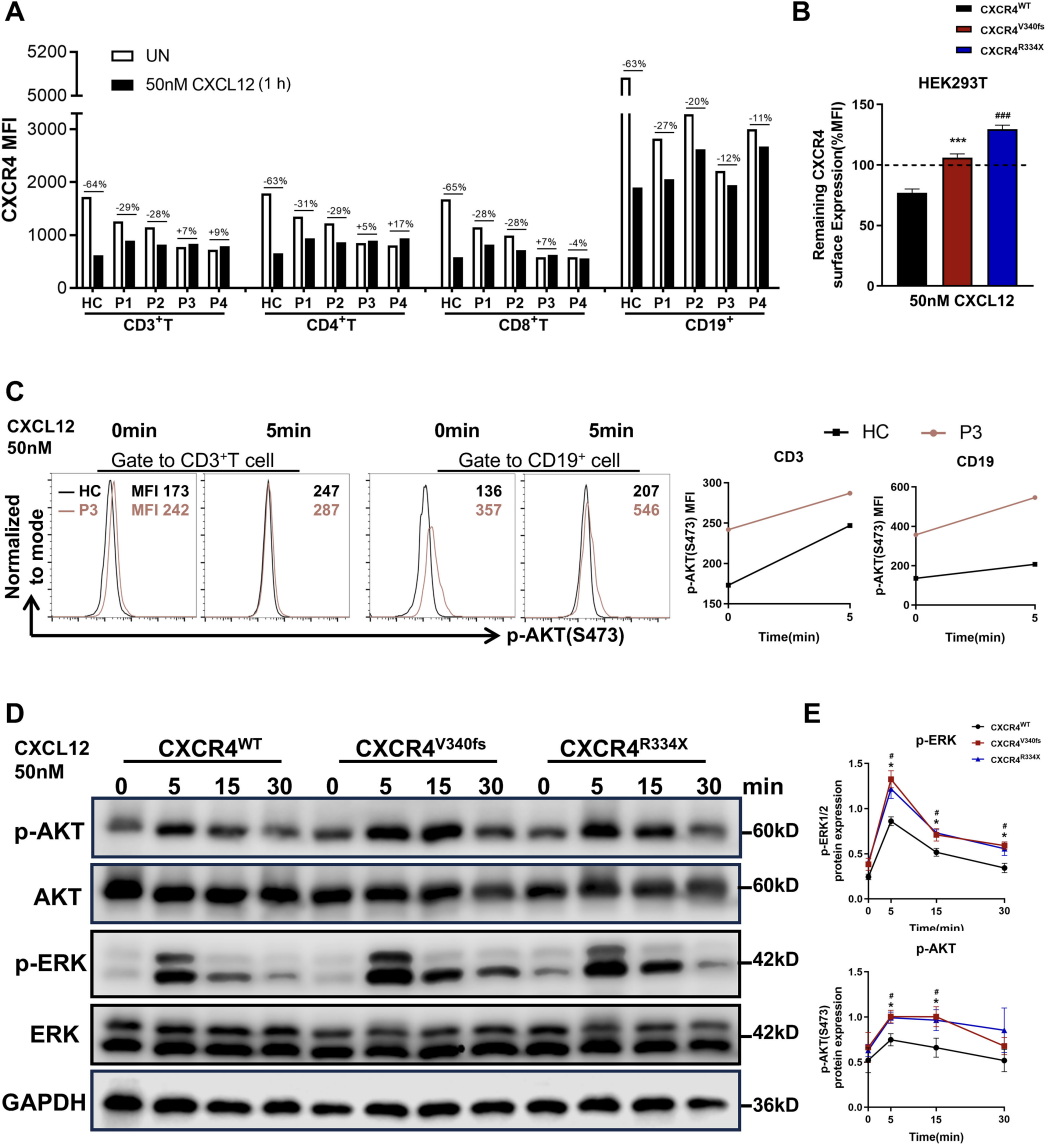
CXCR4^V340fs^ is a gain-of-function mutation. **(A)** Surface CXCR4 expression of PBMCs were measured by FACS using CXCR4-specific mAb 12G5 in the presence or absence of 50nM CXCL12 stimuli. Values represent the percentage of CXCR4 downregulation after stimulation. **(B)** Transiently transfected HEK293T cells were stimulated with vehicle or 50nM CXCL12 for 1h, surface expression of CXCR4 was detected (n=3). The dashed lines represent CXCR4 expression normalized to 100% before stimulation. **(C)** PBMCs from HC and P3 were stimulated with 50nM CXCL12 for 0 or 5 min, fixed, and analyzed for intracellular MFI of p-AKT (S473) by gating on T cells and B cells. **(D)** Transiently transfected HEK293T cells were stimulated with 50nM CXCL12 for the indicated times, and the whole-cell lysates were measured by Western blot to determine total ERK1/2, AKT and p-ERK1/2, p-AKT, GAPDH levels. **(E)** The p-AKT and p-ERK1/2 expression were calculated by band densitometry using ImageJ. Data are shown as mean ± SEM from three independent experiments. *p<0.05, ***p<0.001 for the comparison between CXCR4^V340fs^ and CXCR4^WT^, ^#^ *p*<0.05 and ^###^ *p*<0.001 for the comparison between CXCR4^R334X^ and CXCR4^WT^.

A defect in CXCL12-mediated receptor internalization is one of the primary hallmarks of WHIM syndrome (3, 23, 24). Given the necessity of C-tail integrity for the internalization of CXCR4, we investigated the impact of the CXCR4^V340fs^ variant on this process. Notably, the cell surface expression of CXCR4 was markedly downregulated in PBMCs from HCs following stimulation with CXCL12. In contrast, receptor downregulation in PBMCs from P1–P4 was significantly diminished in response to CXCL12 (**Figure 4A**). Similar trends were observed when cells were treated with varying concentrations of CXCL12. Although P1-P4 all shared the same V340L variant and lived in comparable environment, differences in CXCR4 internalization were noted between P1-P2 and P3-P4 when stimulated with high concentrations of CXCL12 (**Supplementary Figure SB**). To further verify these findings, we constructed plasmids for CXCR4^WT^, CXCR4^V340fs^, and CXCR4^R334X^ and transfected them into HEK293T cells. Cells transfected with CXCR4^WT^ plasmid exhibited downregulation of cell surface CXCR4 following stimulation with CXCL12. Conversely, HEK293T cells transfected with the CXCR4^V340fs^ and CXCR4^R334X^ plasmids showed minimal downregulation (**Figure 4B**), and no significant downregulation was observed even with prolonged stimulation (**Supplementary Figure SC**). Additionally, enhanced migration is a characteristic feature of immune cells expressing CXCR4^WHIM^ receptors. To assess this, we performed a transwell migration assay using HEK293T cells, revealing that the migration of cells expressing the CXCR4^V340L^ and hotspot CXCR4^R334X^ variants was significantly increased (**Supplementary Figure SD**).

CXCR4 is known to promote the phosphorylation of ERK1/2 and AKT through the Gi and GRK/β-arrestin-dependent pathways (25, 26). Previous studies have indicated that most *CXCR4* variants amplify both the amplitude and duration of ERK1/2 and AKT activation in response to CXCL12 stimulation (24). Accordingly, we assessed the expression of p-AKT using flow cytometry and found that the MFI of p-AKT in CD3^+^T cells and B cells from P3 was significantly higher, both before and after CXCL12 stimulation, compared to that in HCs (**Figure 4C**). In parallel, we transfected WT CXCR4 and variant plasmids, specifically CXCR4^V340fs^ and CXCR4^R334X^, into HEK293T cells. The expression levels of p-AKT and p-ERK1/2 following CXCL12 stimulation were found to be stronger and more sustained in cells transfected with the variant plasmids than in those transfected with the CXCR4^WT^ plasmids (**Figures 4D, E**). Thus, we conclude that CXCR4^V340fs^ represents a gain-of-function mutation that impairs receptor internalization, leading to hyperactive downstream signaling following ligand binding.

### Abnormal distribution of lymphocyte subsets in WHIM patients

Additionally, we analyzed blood lymphocyte subsets in all five patients. We noted that the absolute numbers of CD8^+^T cell subsets, particularly naïve CD8^+^T cells, were markedly lower than normal levels in all patients. Furthermore, the number of naïve CD4^+^T cells in P2–P5 was reduced. Consequently, the overall number of T cells was decreased across all patients. Similarly, the counts of B cell subsets in these five patients were significantly lower compared to those in age- and sex-matched controls. In particular, T and B cell subset numbers in P5 were lower than those in P1–P4. The number of NK cells in P2–P4 was also decreased (**Table 3**).

**Table 3:** Analysis of lymphocyte subsets in the peripheral blood.

Parameters	P1 (5y9mo)		P2 (7y10mo)		P3 (35y6mo)		P4 (30y4mo)		P5 (14y)	
	Relative (%)	Absolute (cell/μl)	Relative (%)	Absolute (cell/μl)	Relative (%)	Absolute (cell/μl)	Relative (%)	Absolute (cell/μl)	Relative (%)	Absolute (cell/μl)
**T cell**	68.26 (60.05-74.08)	**1228.68↓** (1424-2664)	**82.37↑** (59.50-75.56)	**1169.65↓** (1480.28-2847.32)	**79.16↑** (61.29-73.13)	**438.55↓** (1169.44-2071.34)	**88.64**↑ (56.84-75.02)	**975.04↓** (1184-2144)	**52.3↓** (56.84-75.02)	**444.6↓** (1184-2144)
**CD8^+^T cell**	**18.8↓** (19.68-34.06)	**338.4↓** (518-1125)	31.58 (19.70-32.04)	**448.44↓** (552.62-1127.28)	**17.71↓** (20.99-33.73)	**98.11↓** (422.79-899.74)	**39.45↑** (21.91-36.80)	**433.95↓** (489-1009)	**17.1↓** (21.91-36.80)	**145.1↓** (489-1009)
**CD8 Naïve**	48.2 (41.58-77.90)	**163.11↓** (297-730)	**34.5↓** (38.03-79.08)	**154.71↓** (293.36-768.42)	**14↓** (37.00-69.35)	**13.74↓** (209.68-560.05)	**25.7↓** (35.34-72.32)	**111.53↓** (231-568)	**9.4↓** (35.34-72.32)	**13.6↓** (231-568)
CD8 TEMRA	10.8 (1.70-24.62)	36.55 (11-218)	27.2↑ (1.30-22.85)	121.97 (9.05-209.78)	48.7↑ (3.90-27.25)	47.78 (20.41-218.45)	59↑ (5.08-31.24)	256.03 (29-269)	8.1 (5.08-31.24)	11.7↓ (29-269)
**CD8 CM**	**32.5↑** (12.08-30.54)	109.98 (85-268)	28.5 (11.91-36.87)	127.8 (79.59-350.41)	30.6 (14.00-36.85)	**30.02↓** (86.65-274.87)	12.3 (10.96-31.00)	**53.38↓** (74-228)	**72.1↑** (10.96-31.00)	104.6 (74-228)
**CD8 EM**	8.55 (1.58-13.18)	28.93 (10-129)	9.78 (1.11-14.51)	43.86 (7.90-104.18)	6.69 (2.40-15.50)	**6.56↓** (12.98-110.27)	3.02 (2.38-15.84)	**13.11↓** (16-109)	10.5 (2.38-15.84)	**15.2↓** (16-109)
**CD4^+^T cell**	**46.92↑** (26.17-40.76)	844.56 (686-1358)	**47.56↑** (28.49-41.07)	**675.35↓** (767.26-1592.48)	**59.06↑** (26.36-40.90)	**327.19↓** (553.66-1108.59)	**48.05↑** (22.25-39.00)	528.55 (522-1084)	25.6 (22.25-39.00)	**217.8↓** (522-1084)
**CD4 Naïve**	67.7 (45.56-75.28)	571.77 (321-972)	49.7 (40.75-72.70)	**335.65↓** (338.68-1036.97)	**14.8↓** (43.30-63.20)	**48.42↓** (276.06-653.56)	**23.1↓** (39.50-66.26)	**122.1↓** (230-627)	**18.1↓** (39.50-66.26)	**39.4↓** (230-627)
**CD4 TEMRA**	0.31 (0.00-1.06)	2.62 (0-13)	0.39 (0.00-1.47)	2.63 (0.00-16.71)	0.57 (0.10-2.10)	1.86 (0.52-18.20)	0.85 (0.00-1.54)	4.49 (0-12)	1.2 (0.00-1.54)	2.7 (0-12)
**CD4 CM**	28.5 (22.06-46.46)	240.7 (211-478)	46.5 (21.66-52.74)	314.04 (232.09-600.93)	**71.6↑** (30.85-45.25)	234.27 (202.98-421.64)	**62.6↑** (25.34-49.90)	330.87 (182-403)	**56.1**↑ (25.34-49.90)	**122.2↓** (182-403)
**CD4 EM**	3.5 (2.08-8.78)	29.56 (23-84)	3.39 (1.90-9.20)	22.89 (20.54-96.75)	13.1 (4.20-16.25)	42.86 (31.06-127.52)	13.5 (4.68-15.70)	71.35 (29-117)	24.6↑ (4.68-15.70)	53.6 (29-117)
**CD4:CD8**	**2.5↑** (0.87-1.94)	–	1.51 (1.02-2.05)	–	**3.33↑** (0.85-1.76)	–	1.22 (0.65-1.65)	–	1.5 (0.65-1.65)	–
**TCRαβ^+^DNT**	0.93 (0.18-2.81)	11.49 (4-55)	1.02 (0.19-2.43)	11.91 (3.77-49.48)	1.23 (0.80-2.52)	**5.39↓** (13.16-43.77)	0.78 (0.61-2.31)	**7.61↓** (12-37)	1.5 (0.61-2.31)	**6.7↓** (12-37)
**γδ T cell**	14.1 (6.92-19.84)	173.24 (124-410)	2.02 (7.00-19.60)	**23.63↓** (133.70-427.77)	**24.8↑** (6.40-18.50)	108.76 (85.12-358.12)	**27.6↑** (6.55-20.28)	269.11 (81-343)	**21.9↑** (6.55-20.28)	97.4 (81-343)
**NK cell**	26.14↑ (9.00-22.24)	470.52 (258-727)	9.99 (7.83-20.99)	**141.86↓** (227.47-667.76)	17.01 (11.43-27.57)	**94.24↓** (231.75-788.80)	**8.35↓** (10.12-28.34)	**91.85↓** (210-804)	**44.3↑** (10.12-28.34)	376.3 (210-804)
**B cell**	**5.18↓** (10.21-20.12)	**93.24↓** (280-623)	**7.62↓** (10.46-21.77)	**108.2↓** (303.52-777.25)	**3.74↓** (7.73-16.84)	20.72↓ (176.56-415.64)	**2.99↓** (8.84-17.76)	**32.89↓** (203-476)	**2.1↓** (8.84-17.76)	**17.5↓** (203-476)
**Memory B**	**5.05↓** (7.76-19.90)	**4.71↓** (31-94)	**5.97↓** (8.61-20.19)	**6.46↓** (37.69-114.81)	**27.2↑** (8.94-23.75)	5.64↓ (26.66-70.28)	13.5 (7.15-23.10)	**4.44↓** (20-86)	**6.7↓** (7.15-23.10)	**1.2↓** (20-86)
**Naïve B**	**89↑** (48.36-75.84)	**82.98↓** (147-431)	**81.7↑** (52.04-75.78)	**88.4↓** (171.45-469.28)	60.7 (48.38-77.85)	**12.58↓** (98.18-296.15)	75.6 (53.78-78.64)	**24.86↓** (116-347)	**83.8↑** (53.78-78.64)	**14.7↓** (116-347)
**Transitional B**	6.5 (2.58-12.30)	**6.06↓** (10-66)	9.28 (3.41-11.17)	**10.04↓** (14.36-59.62)	**6.85↑** (1.35-5.50)	**1.42↓** (3.14-18.90)	**11.8↑** (1.38-9.42)	**3.88↓** (4-37)	5.2 (1.38-9.42)	**0.9↓** (4-37)
**Plasmablasts B**	4.03 (0.90-7.36)	**3.76↓** (4-28)	1.92 (0.80-9.75)	**2.08↓** (3.87-39.83)	**9.19↑** (0.46-5.80)	1.9 (1.25-14.63)	2.57 (0.49-7.06)	**0.85↓** (1-23)	1.6 (0.49-7.06)	**0.3↓** (1-23)
**Neutrophil**	40.9 (33-79)	**970↓** (1600-7800)	37 (33-79)	**740↓** (1600-7800)	**33.4↓** (45-77)	**410↓** (2000-7000)	**23.9↓** (40-75)	**510↓** (1800-6300)	**19.2↓** (37-77)	**150↓** (1800-8300)

CM, central memory; EM, effector memory; TEMRA, terminally differentiated effector memory T lymphocytes. The values in brackets are normal reference. The values in bold indicate abnormal values. ↓ represent decreased, ↑ represent increased.

**Figure 5:**
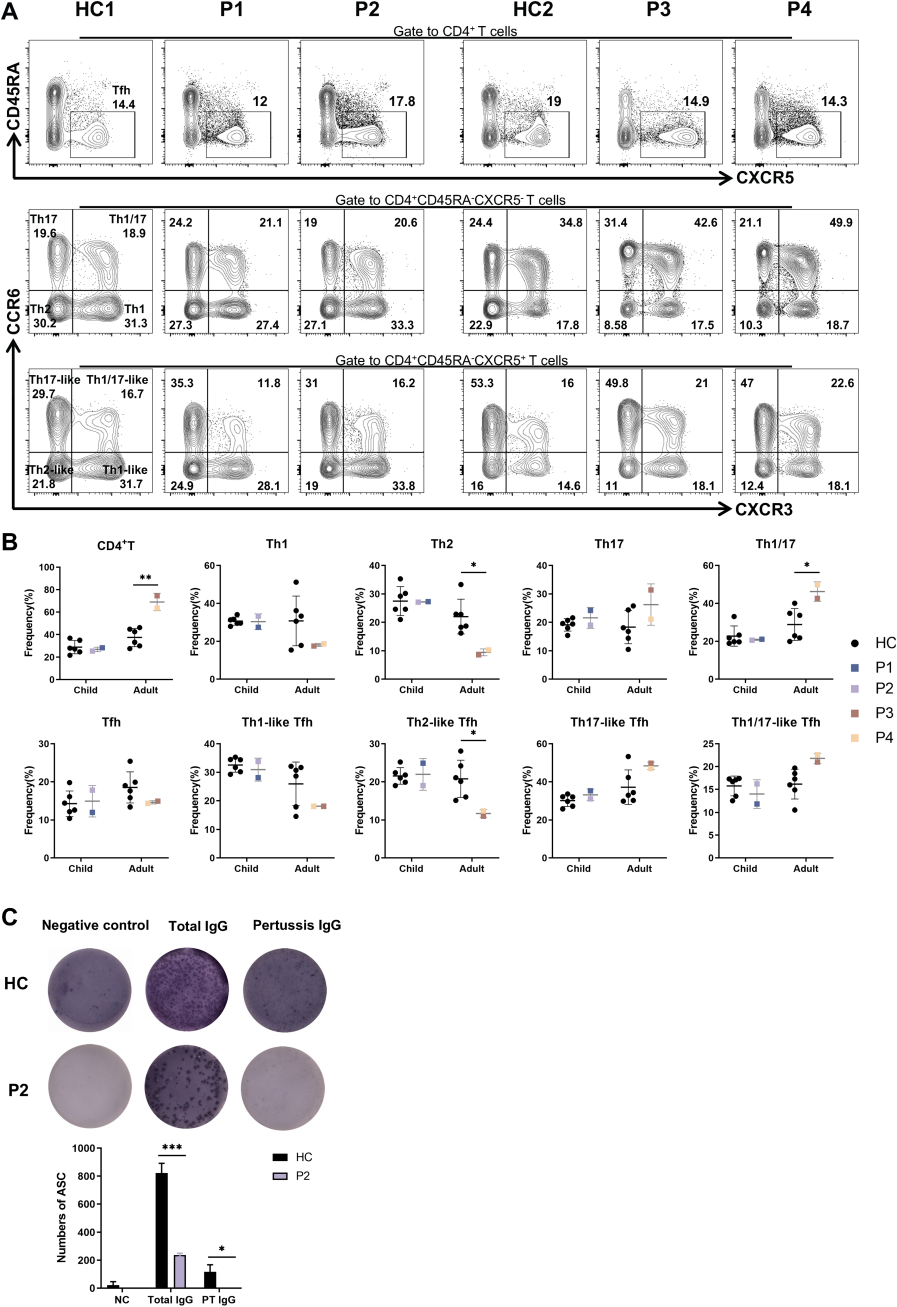
Abnormal distributions of lymphocyte subsets and impaired ability to produce PT specific IgG in patients. **(A)** The percentages of Tfh, Th1-like, Th2-like, Th17-like, Th1/17-like and Th1, Th2, Th17, Th1/17 cells in patients and six age-matched HCs. **(B)** Statistics of CD4^+^T and Tfh cell subpopulations in panel **(A)**. **(C)** Representative images of total IgG and PT specific IgG detected by ELISPOTs in P2 and HC. *P<0.05, ***P<0.001 for comparison between patients and HCs.

We next evaluated the proportion of CD4^+^T (Th) and T follicular helper (Tfh) subsets in P1–P4. Notably, P3 and P4 showed an increased frequency of Th and Th1/17 cells, while the frequency of Th2 and Th2-like Tfh cells was reduced. In contrast, the proportions of Th and Tfh cell subsets in P1 and P2 were comparable to those observed in HCs (**Figures 5A, B**).

Given that none of the patients in family 1 exhibited hypogammaglobulinemia, we performed ELISpot assays to assess lgG secretion by memory B cells. P1 and P2 had been vaccinated according to the Childhood Immunization Schedule for National Immunization Program Vaccines - China. According to this program and P2’s vaccination record, P2 received DTaP vaccination (diphtheria, tetanus, and pertussis) at 3, 4, 5 and 18 months of age. Since the DTaP vaccine is provided free of charge and is mandatory for all individuals in our country, and given that pertussis toxin (PT) is one of the immunogens available for purchase, we specifically tested for PT-specific IgG production using the ELISpot assay. Compared to cells from the HC, cells from P2 exhibited a diminished ability to produce PT-specific IgG (**Figure 5C**). Overall, theses data suggest that the CXCR4^V340fs^ variant adversely affects the immune functions of both T and B cells.

## Discussion

WHIM syndrome was first described in 1964 (27); while heterozygous mutations in the chemokine receptor CXCR4 identified as the cause in 2003 (4). The classic clinical manifestation is tetralogy; however, even the hotspot mutation (CXCR4^R334X^) may present with atypical clinical manifestations, which only presented with myelokathexis (28). Furthermore, patients within the same family who share the same genotype can exhibit varying clinical symptoms (29). Thus, the considerable heterogeneous in the age of diagnosis and clinical manifestations complicate the identification of this syndrome. In this study, we provide a detailed clinical, genetic and immunological characterization of four WHIM patients from two generations of a Chinese family, all of whom harbor an extended mutation caused by a pair of base duplications (c.1016_1017dupCT, p.V340Lfs*27) in *CXCR4* gene. Compared to reported patients with the CXCR4^R334X^ mutation, these four individuals with the CXCR4^V340fs^ mutation exhibit atypical phenotypes.

The clinical manifestations of WHIM syndrome are notably heterogeneous (10); indeed, only about one-third of patients meet the diagnostic tetrad criteria for WHIM syndrome. The majority of patients experience non-cyclic neutropenia (98%), followed by lymphopenia (88%) and hypogammaglobulinemia (65%). Recurrent infections occur in 92% of patients, predominantly presenting as otitis media and pneumonia, while approximately 40% of patients develop HPV-related warts on various body parts (9). None of the patients in family 1 exhibited all four features of WHIM syndrome; In contrast, P5 from family 2 presented with warts, hypogammaglobulinemia, recurrent infections, and myelokathexis, along with bronchiectasis and conductive deafness resulting from recurrent infections. Given the differences in clinical manifestations between the patients in Family 1 and P5, we conducted a literature search for patients with similar CXCR4 mutations as those in Family 2 (**Supplementary Table S3**). As indicated in **Table 2**, (a) Despite being in their thirties, neither P3 nor P4 has experienced bronchiectasis or hearing loss due to recurrent infections; (b) P4 had one wart on his hand, while no warts were observed in P1, P2, or P3. Conversely, multiple warts were found in all adult and older children with CXCR4^R334X^ mutation. This suggests that HPV infection in our four patients is relatively mild. (c) In terms of treatment, none of our four patients received antibiotic prophylaxis, G-CSF, Regular IgGRT, Plerixafor, HSCT, etc. They are currently in asymptomatic condition and have no severe infections occurred. In summary, as P1 and P3 only presented with one typical symptom (myelokathexis), P4 presented with warts, we can infer that the four WHIM patients we reported presented atypical clinical phenotypes. Notably, P2 also experienced nephrotic syndrome, though no other related renal diseases have been reported in the past, aside from congenital renal anomalies. However, Previous studies have indicated that CXCR4 plays a role in kidney fibrosis through multiple effectors (30). Additionally, CXCR4 has been shown to induce podocyte injury and proteinuria by activating β-catenin signaling (31). Consequently, it remains unclear whether the genetic mutation or WHIM syndrome contributes to renal disease, necessitating further investigation through additional cases and longitudinal follow-up. Research suggests that the heterogeneity of clinical features observed in WHIM patients may be linked to mutant CXCR4-dependent alterations in signaling pathways (29). However, due to the significant clinical heterogeneity of primary immunodeficiency disease and the limited number of WHIM patients, the relationship between the CXCR4^V340fs^ variant and milder clinical phenotypes requires further exploration through larger case studies.

Furthermore, several case reports have documented variants occurring near amino acid 340 of the CXCR4 protein. One notable study described a truncated mutation (S339fsX342) resulting from a c.1016_1017 deletion at the C-terminus, which led to the full tetrad of WHIM syndrome features. This patient additionally experienced persistent enteritis and received treatment with G-CSF, subcutaneous immunoglobulin, and antibiotics. Notably, the clinical manifestations of this WHIM patient with the extended mutation (S341fsX365) were strikingly similar to those of P1–P4, including warts, neutropenia, recurrent respiratory infections, and myelokathexis; however, hypogammaglobulinemia was not observed. Moreover, the patient only received IVIG treatment for 3 years during childhood (32). A recently reported case of WHIM (S339L fs*27) presented with myelokathexis and recurrent infections, without warts or hypogammaglobulinemia (9). Overall, the relationship between the clinical phenotype of WHIM syndrome and mutations needs to be further verified.

CXCR4 is a 7-transmembrane G protein-coupled receptor that binds CXCL12. The cytoplasmic C-terminal tail CXCR4 contains a series of phosphorylation motifs that regulate downstream signaling, β-arrestin binding, and the receptor’s internalization. Impaired internalization and heightened signaling responsiveness are characteristic features of the CXCR4^WHIM^ receptors. Our study, along with previously published research, has demonstrated that CXCR4^WHIM^ mutations result in diminished receptor internalization in response to CXCL12 stimulation. In our cohort, all four patients exhibited varying degrees of impaired CXCR4 internalization; pediatric patients showed reduced internalization, while adult patients demonstrated no internalization. However, previous studies have not extensively analyzed the relationship between age and the degree of CXCR4 internalization. We infer that the observed differences in our study may be attributed to factors such as disease heterogeneity, individual variation, age, CXCR4 expression levels, and environmental influences. Additionally, we found that both cell surface and total CXCR4 expression levels were higher in pediatric patients compared to adult patients. Assays measuring cellular chemotaxis in response to CXCL12, intracellular calcium mobilization, and PI3K-Akt/extracellular signal-regulated kinase activation have also been frequently utilized in our investigations. In our study we observed that the expression of p-AKT and p-ERK1/2 was elevated following CXCL12 stimulation, indicating hyperactive downstream signaling. However, some studies have reported no enhancement in the phosphorylation of AKT and ERK1/2 (29). Additionally, calcium mobilization responses varied among patients, with some showing increased levels while others demonstrated decreased levels (11, 12, 24, 33). These disparate outcomes within the same signaling pathway may contribute to the clinical heterogeneity observed among WHIM patients.

The CXCR4/CXCL12 axis is crucial for bone marrow colonization during development and for hematopoietic stem cell homeostasis (34). Meanwhile, CXCR4 plays a vital role in orchestrating both innate and adaptive immune responses by regulating the transport and distribution of leukocytes from peripheral tissues, as well as by enhancing T cell priming through its contribution to the formation and stability of immune synapses (35, 36). While circulating T cell counts may be reduced in WHIM patients, as previously reported, the decrease is less pronounced compared to that of B cells and neutrophils. CD4^+^T cell counts may remain within the reference range or exhibit slight reductions, whereas CD8^+^T numbers appear to be more adversely affected. Furthermore, the numbers of naïve T cell subset are diminished, likely due to reduced thymic output (22, 37, 38). In our findings, we also noted a decrease in TRECs in P1, P2 and P5. Additionally, the counts of naïve T cell, particularly naïve CD8^+^T cell, in the peripheral blood of the patients were significantly reduced. Whether this result was associated with decreased expression of CXCR4 on the surface of CD8^+^T cells rather than CD4^+^T cells requires further research.

CXCR4 is essential for the homing, development, and function of B cells. Throughout all stages of development, B cells typically express CXCR4 at markedly higher levels than most other leukocyte subsets (6, 39). Targeted deletion of *CXCR4* in B cells results in premature egress of B cell precursors from the bone marrow and subsequent localization within splenic follicles, leading to a reduction in the number of mature B cells in the splenic marginal zone and primary follicles, as well as impaired T-independent antibody responses (40, 41). WHIM patients exhibit a diminished capacity to generate hypermutated IgG and an inability to initiate antigen-specific memory responses (39). In our study, we found that CXCR4 expression on the surface of B cells from all patients was significantly lower than that in HCs. Moreover, the number of B cell subsets was markedly reduced in our patients, with P5displaying the lowest count of B cell subsets. Additionally, KRECs were decreased in P1, P2 and P5. Furthermore, the production of PT-specific IgG by memory B cells from P2 was also reduced. While a reduction in B cells is a prominent hematological feature among WHIM patients (42), the mechanisms by which patients with severe B lymphopenia avoid hypogammaglobulinemia remain unclear.

WHIM syndrome is a relatively benign IEI characterized by low mortality rates. Currently, there is no standardized treatment protocol; the primary objective is to prevent HPV-related malignant and chronic sequelae resulting from recurrent infections (e.g., bronchiectasis and hearing loss). It is widely accepted that regular IVIG infusion and prophylactic antibiotics can help prevent recurrent respiratory and ear infections. G-CSF has proven effective in mobilizing neutrophils from the bone marrow into the bloodstream (10, 43). A completed phase III clinical trial of the CXCR4 antagonist plerixafor confirmed its superiority over G-CSF with in terms of wart regression and hematologic improvement (44). Additionally, a phase III trial of mavorixafor demonstrated its efficacy in increasing neutrophil and lymphocyte counts while alleviating infection (45). Regrettably, with the exception of irregular IgGRT, none of the patients P1-P4 received any additional treatments and are currently asymptomatic. Consequently, regular follow-up for these patients is essential.

In conclusion, we have detailed the clinical, genetic, immunological and treatment characteristic of four patients with the CXCR4^V340fs^ WHIM mutation from a single Chinese family who exhibited atypical phenotypes. Given the high heterogeneity associated with WHIM syndrome, this study contributes to expanding the understanding of its clinical spectrum.

## Data Availability

The datasets generated during and/or analyzed during the current study are available from the corresponding author on reasonable request.
