# Corrigendum: Heterogeneous phenotype of a Chinese Familial WHIM syndrome with CXCR4^V340fs^ gain-of-function mutation

**DOI:** 10.3389/fimmu.2024.1544964

**Published:** 2025-01-14

**Authors:** Yu Huang, Lu Li, Ran Chen, Lang Yu, Shunkai Zhao, Yanjun Jia, Ying Dou, Zhiyong Zhang, Yunfei An, Xuemei Tang, Xiaodong Zhao, Lina Zhou

**Affiliations:** ^1^ National Clinical Research Center for Child Health and Disorders, Ministry of Education Key Laboratory of Child Development and Disorders, Children’s Hospital of Chongqing Medical University, Chongqing, China; ^2^ Chongqing Key Laboratory of Child Rare Diseases in Infection and Immunity, Children’s Hospital of Chongqing Medical University, Chongqing, China; ^3^ Department of Hematology Oncology, Children’s Hospital of Chongqing Medical University, Chongqing, China; ^4^ Department of Biology, School of Arts and Sciences, Tufts University, Medford, MA, United States; ^5^ Department of Rheumatism and Immunology, Children’s Hospital of Chongqing Medical University, Chongqing, China

**Keywords:** CXCR4 variant, gain-of-function, inborn error of immunity, WHIM syndrome, heterogeneous phenotype

In the published article, there was an error. A typo was made.

A correction has been made to **Abstract,** *Results*, Paragraph 1. This sentence previously stated:

“We provide and in-depth analysis of their clinical, genetic, immunological and treatment characteristic, noting that these patients exhibited an atypical clinical phenotype when compared to reported CXCR4R334X patients.”

The corrected sentence appears below:

“We provide an in-depth analysis of their clinical, genetic, immunological and treatment characteristic, noting that these patients exhibited an atypical clinical phenotype when compared to reported CXCR4R334X patients.”

1. In the published article, there was an error. A typo was made.

A correction has been made to **Introduction**, Paragraph 2. This sentence previously stated:

“Thirty-seven distinct CXCR4 variants have been identified, whice including eight nonsense variants, twenty-seven frameshift variants, and two missense variants.”

The corrected sentence appears below:

Thirty-seven distinct CXCR4 variants have been identified, which including eight nonsense variants, twenty-seven frameshift variants, and two missense variants.”

2. In the published article, there was an error. Omission of important information.

A correction has been made to **Results**, *Clinical manifestations of the family with WHIM syndrome*, Paragraph 3. This sentence previously stated:

“At the age of, P2 was hospitalized due to edema, and subsequent tests showed proteinuria, hyperlipidemia, and hypoproteinemia, leading to a diagnosis of nephrotic syndrome.”

The corrected sentence appears below:

“At the age of 4, P2 was hospitalized due to edema, and subsequent tests showed proteinuria, hyperlipidemia, and hypoproteinemia, leading to a diagnosis of nephrotic syndrome.”

3. In the published article, there was an error. A typo was made.

A correction has been made to **Results**, *Decreased surface CXCR4 expression on CD8+T cells and B cells from patients*, Paragraph 1. This sentence previously stated:

“Interestingly, P1-P4 exhibited relatively higher levels of CXCR4 lexpression, particularly in pediatric patients P1 and P2 (**Supplementary Figure SA**).”

The corrected sentence appears below:

“Interestingly, P1-P4 exhibited relatively higher levels of CXCR4 expression, particularly in pediatric patients P1 and P2 (**Supplementary Figure SA**).”

4. In the published article, there was an error. A typo was made.

A correction has been made to **Discussion**, Paragraph 4. This sentence previously stated:

“n our cohort, all four patients exhibited varying degrees of impaired CXCR4 internalization; pediatric patients showed reduced internalization, while adult patients demonstrated no internalization.”

The corrected sentence appears below:

“In our cohort, all four patients exhibited varying degrees of impaired CXCR4 internalization; pediatric patients showed reduced internalization, while adult patients demonstrated no internalization.”

The authors apologize for these errors and state that this does not change the scientific conclusions of the article in any way. The original article has been updated.

