# Supplementary figures and images for: Heterogeneous phenotype of a Chinese Familial WHIM syndrome with CXCR4V340fs gain-of-function mutation
Source: Front Immunol. 2024 Nov 7;15:1460990. doi: 10.3389/fimmu.2024.1460990 (PMC11578956; doi:10.3389/fimmu.2024.1460990)

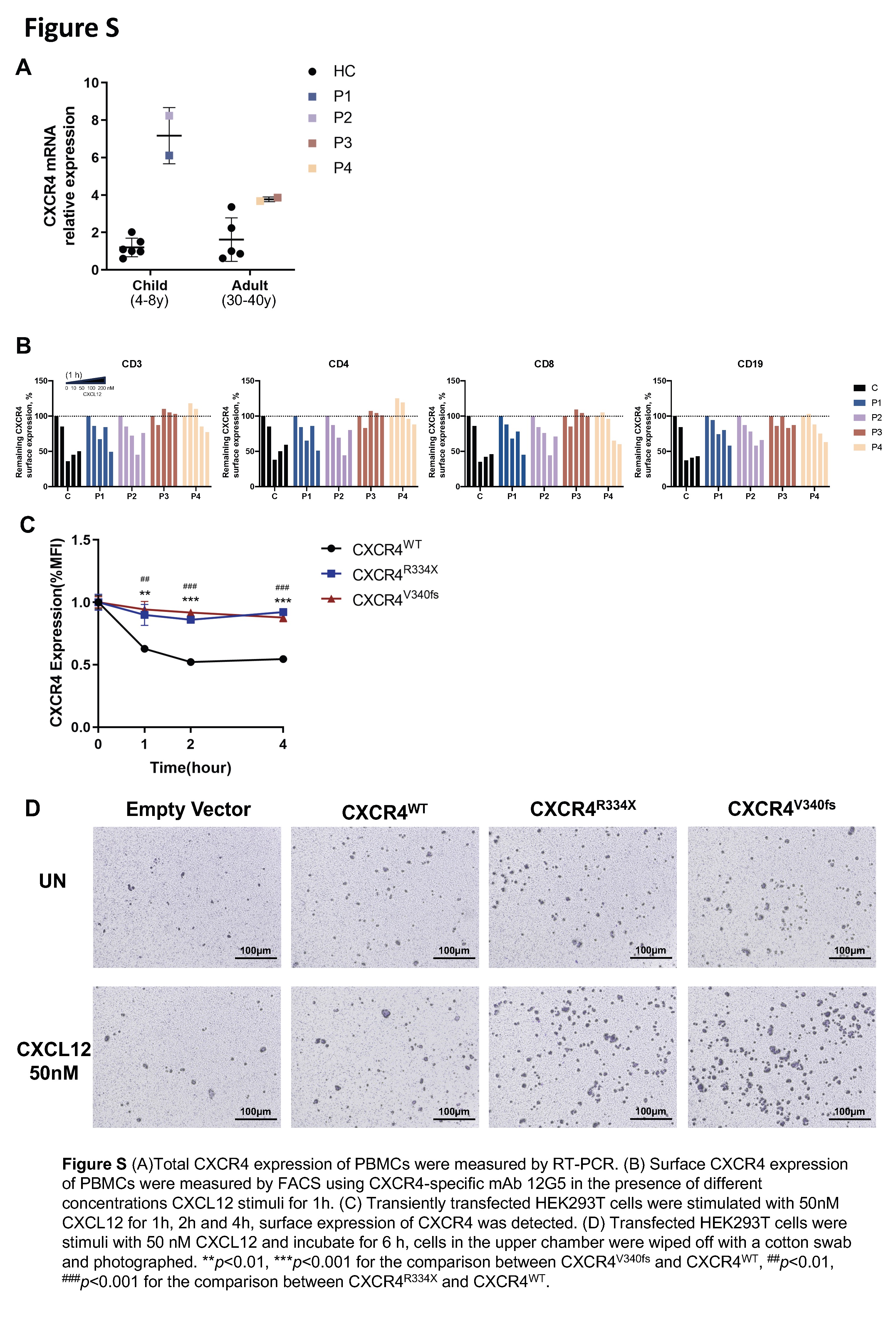

Supplement: Supplementary file 1 [file Image1.tiff]
