# Supplementary material for: Heterogeneous phenotype of a Chinese Familial WHIM syndrome with CXCR4V340fs gain-of-function mutation
Source: Front Immunol. 2024 Nov 7;15:1460990. doi: 10.3389/fimmu.2024.1460990 (PMC11578956; doi:10.3389/fimmu.2024.1460990)
Supplement: Supplementary file 2 [file Table1.docx]

**Supplementary Table Comparison of hotspot mutation R334X with P1-P4 in detail**

| **Reference** | |  | (1, 2) | | (3) | (4) | (5) | (6) | | (7) | (8) | (9) | (10) | (11) | | | | (12) | | | | (13) | | | (14) | (15, 16) | (17, 18) | | (19, 20) | |  |  |  |  |
| --- | --- | --- | --- | --- | --- | --- | --- | --- | --- | --- | --- | --- | --- | --- | --- | --- | --- | --- | --- | --- | --- | --- | --- | --- | --- | --- | --- | --- | --- | --- | --- | --- | --- | --- |
| **Patient** | | **P5** | Control | | | | | | | | | | | | | | | | | | | | | | | | | | | | **P1** | **P2** | **P3** | **P4** |
| **Gender** | | **M** | F | F | M | F | F | M | M | F | F | M | F |  |  |  |  | F | M | F | F | F | M | M |  | F | F | F | F | M | **M** | **F** | **F** | **M** |
| **Mutation** | DNA | **1000C>T** | | | | | | | | | | | | | | | | | | | | | | | | | | | | | **1016_1017dupCT** | | | |
|  | Amino acid | **R334X** | | | | | | | | | | | | | | | | | | | | | | | | | | | | | **V340Lfs*27** | | | |
| **Age at onset** | | **3y** | 6mo | 6mo | 8y | birth | birth | childhood |  |  | 6mo | 12y |  | 1.9y | 0.3y | 0.3 | 9y | 5 | Child | 2 | Born | 2mo | 14d | birth | Infancy | 2 | Infancy | 1.5 | 7 | 29 | **3mo** | **4y** | **24y** |  |
| **Age at diagnosis** | | **11y** | 5y7mo | 5y7mo | 23y | 5y | 10mo | 30y | 51y | 4y | 5y | 32y | 12y | 7y | 14y | 4.5 | 9y |  |  |  |  | 25y | 8mo | 4mo | 52y |  |  |  |  |  | **4y** | **7y** | **33y** | **31y** |
| **Respiratory symptoms** | RRI |  |  |  |  |  |  |  |  |  |  |  |  |  |  |  |  |  |  |  |  |  |  |  |  |  |  |  |  |  |  |  |  |  |
|  | Sinusitis |  |  |  |  |  |  |  |  |  |  |  |  |  |  |  |  |  |  |  |  |  |  |  |  |  |  |  |  |  |  |  |  |  |
|  | Pneumonia |  |  |  |  |  |  |  |  |  |  |  |  |  |  |  |  |  |  |  |  |  |  |  |  |  |  |  |  |  |  |  |  |  |
|  | Atelectasis |  |  |  |  |  |  |  |  |  |  |  |  |  |  |  |  |  |  |  |  |  |  |  |  |  |  |  |  |  |  |  |  |  |
|  | Bronchiectasis |  |  |  |  |  |  |  |  |  |  |  |  |  |  |  |  |  |  |  |  |  |  |  |  |  |  |  |  |  |  |  |  |  |
| **Skin infections** | Cellulitis |  |  |  |  |  |  |  |  |  |  |  |  |  |  |  |  |  |  |  |  |  |  |  |  |  |  |  |  |  |  |  |  |  |
|  | Abscesses |  |  |  |  |  |  |  |  |  |  |  |  |  |  |  |  |  |  |  |  |  |  |  |  |  |  |  |  |  |  |  |  |  |
|  | Folliculitis |  |  |  |  |  |  |  |  |  |  |  |  |  |  |  |  |  |  |  |  |  |  |  |  |  |  |  |  |  |  |  |  |  |
|  | Cutaneous ulcers |  |  |  |  |  |  |  |  |  |  |  |  |  |  |  |  |  |  |  |  |  |  |  |  |  |  |  |  |  |  |  |  |  |
|  | HSV |  |  |  |  |  |  |  |  |  |  |  |  |  |  |  |  |  |  |  |  |  |  |  |  |  |  |  |  |  |  |  |  |  |
|  | VZV |  |  |  |  |  |  |  |  |  |  |  |  |  |  |  |  |  |  |  |  |  |  |  |  |  |  |  |  |  |  |  |  |  |
|  | Rubella |  |  |  |  |  |  |  |  |  |  |  |  |  |  |  |  |  |  |  |  |  |  |  |  |  |  |  |  |  |  |  |  |  |
| **Oral** | Gingivitis |  |  |  |  |  |  |  |  |  |  |  |  |  |  |  |  |  |  |  |  |  |  |  |  |  |  |  |  |  |  |  |  |  |
|  | PD |  |  |  |  |  |  |  |  |  |  |  |  |  |  |  |  |  |  |  |  |  |  |  |  |  |  |  |  |  |  |  |  |  |
| **Gastrointestinal Symptoms** | AU |  |  |  |  |  |  |  |  |  |  |  |  |  |  |  |  |  |  |  |  |  |  |  |  |  |  |  |  |  |  |  |  |  |
|  | Enteritis |  |  |  |  |  |  |  |  |  |  |  |  |  |  |  |  |  |  |  |  |  |  |  |  |  |  |  |  |  |  |  |  |  |
| **Autoimmunity** | ITP |  |  |  |  |  |  |  |  |  |  |  |  |  |  |  |  |  |  |  |  |  |  |  |  |  |  |  |  |  |  |  |  |  |
|  | CPAHA |  |  |  |  |  |  |  |  |  |  |  |  |  |  |  |  |  |  |  |  |  |  |  |  |  |  |  |  |  |  |  |  |  |
|  | TD1 |  |  |  |  |  |  |  |  |  |  |  |  |  |  |  |  |  |  |  |  |  |  |  |  |  |  |  |  |  |  |  |  |  |
| **Ear symptom** | Otitis media |  |  |  |  |  |  |  |  |  |  |  |  |  |  |  |  |  |  |  |  |  |  |  |  |  |  |  |  |  |  |  |  |  |
|  | Hearing loss |  |  |  |  |  |  |  |  |  |  |  |  |  |  |  |  |  |  |  |  |  |  |  |  |  |  |  |  |  |  |  |  |  |
| **Warts** | Face |  |  |  |  |  |  |  |  |  |  |  |  |  |  |  |  |  |  |  |  |  |  |  |  |  |  |  |  |  |  |  |  |  |
|  | Hands |  |  |  |  |  |  |  |  |  |  |  |  |  |  |  |  |  |  |  |  |  |  |  |  |  |  |  |  |  |  |  |  |  |
|  | Feet |  |  |  |  |  |  |  |  |  |  |  |  |  |  |  |  |  |  |  |  |  |  |  |  |  |  |  |  |  |  |  |  |  |
|  | Legs |  |  |  |  |  |  |  |  |  |  |  |  |  |  |  |  |  |  |  |  |  |  |  |  |  |  |  |  |  |  |  |  |  |
|  | Genital |  |  |  |  |  |  |  |  |  |  |  |  |  |  |  |  |  |  |  |  |  |  |  |  |  |  |  |  |  |  |  |  |  |
| **Other infections** | Lymphadenitis |  |  |  |  |  |  |  |  |  |  |  |  |  |  |  |  |  |  |  |  |  |  |  |  |  |  |  |  |  |  |  |  |  |
|  | Osteomyelitis |  |  |  |  |  |  |  |  |  |  |  |  |  |  |  |  |  |  |  |  |  |  |  |  |  |  |  |  |  |  |  |  |  |
|  | Conjunctivitis |  |  |  |  |  |  |  |  |  |  |  |  |  |  |  |  |  |  |  |  |  |  |  |  |  |  |  |  |  |  |  |  |  |
|  | VI |  |  |  |  |  |  |  |  |  |  |  |  |  |  |  |  |  |  |  |  |  |  |  |  |  |  |  |  |  |  |  |  |  |
|  | UTI |  |  |  |  |  |  |  |  |  |  |  |  |  |  |  |  |  |  |  |  |  |  |  |  |  |  |  |  |  |  |  |  |  |
| **Congenital abnormalities** | VSD |  |  |  |  |  |  |  |  |  |  |  |  |  |  |  |  |  |  |  |  |  |  |  |  |  |  |  |  |  |  |  |  |  |
|  | Pulmonary atresia |  |  |  |  |  |  |  |  |  |  |  |  |  |  |  |  |  |  |  |  |  |  |  |  |  |  |  |  |  |  |  |  |  |
|  | FT |  |  |  |  |  |  |  |  |  |  |  |  |  |  |  |  |  |  |  |  |  |  |  |  |  |  |  |  |  |  |  |  |  |
|  | CH |  |  |  |  |  |  |  |  |  |  |  |  |  |  |  |  |  |  |  |  |  |  |  |  |  |  |  |  |  |  |  |  |  |
| **Malignancy** | HPV-related cancer |  |  |  |  |  |  |  |  |  |  |  |  |  |  |  |  |  |  |  |  |  |  |  |  |  |  |  |  |  |  |  |  |  |
|  | EBV-driven lymphomas |  |  |  |  |  |  |  |  |  |  |  |  |  |  |  |  |  |  |  |  |  |  |  |  |  |  |  |  |  |  |  |  |  |
|  | CBCC |  |  |  |  |  |  |  |  |  |  |  |  |  |  |  |  |  |  |  |  |  |  |  |  |  |  |  |  |  |  |  |  |  |
|  | ISCC |  |  |  |  |  |  |  |  |  |  |  |  |  |  |  |  |  |  |  |  |  |  |  |  |  |  |  |  |  |  |  |  |  |
|  | Other malignancies |  |  |  |  |  |  |  |  |  |  |  |  |  |  |  |  |  |  |  |  |  |  |  |  |  |  |  |  |  |  |  |  |  |
| **Idiopathic mental retardation** | |  |  |  |  |  |  |  |  |  |  |  |  |  |  |  |  |  |  |  |  |  |  |  |  |  |  |  |  |  |  |  |  |  |
| **Premature delivery** | |  |  |  |  |  |  |  |  |  |  |  |  |  |  |  |  |  |  |  |  |  |  |  |  |  |  |  |  |  |  |  |  |  |
| **Myelokathexis** | |  |  |  |  |  |  |  |  |  |  |  |  |  |  |  |  |  |  |  |  |  |  |  |  |  |  |  |  |  |  |  |  |  |
| **Laboratory examination abnormal** | Non-cyclic neutropenia |  |  |  |  |  |  |  |  |  |  |  |  |  |  |  |  |  |  |  |  |  |  |  |  |  |  |  |  |  |  |  |  |  |
|  | Lymphopenia |  |  |  |  |  |  |  |  |  |  |  |  |  |  |  |  |  |  |  |  |  |  |  |  |  |  |  |  |  |  |  |  |  |
|  | Monocytopenia |  |  |  |  |  |  |  |  |  |  |  |  |  |  |  |  |  |  |  |  |  |  |  |  |  |  |  |  |  |  |  |  |  |
|  | Hypogammaglobulinemia |  |  |  |  |  |  |  |  |  |  |  |  |  |  |  |  |  |  |  |  |  |  |  |  |  |  |  |  |  |  |  |  |  |
| **Treatment** | Antibiotic prophylaxis |  |  |  |  |  |  |  |  |  |  |  |  |  |  |  |  |  |  |  |  |  |  |  |  |  |  |  |  |  |  |  |  |  |
|  | G-CSF |  |  |  |  |  |  |  |  |  |  |  |  |  |  |  |  |  |  |  |  |  |  |  |  |  |  |  |  |  |  |  |  |  |
|  | Regular IgGRT |  |  |  |  |  |  |  |  |  |  |  |  |  |  |  |  |  |  |  |  |  |  |  |  |  |  |  |  |  |  |  |  |  |
|  | Irregular IgGRT |  |  |  |  |  |  |  |  |  |  |  |  |  |  |  |  |  |  |  |  |  |  |  |  |  |  |  |  |  |  |  |  |  |
|  | Plerixafor |  |  |  |  |  |  |  |  |  |  |  |  |  |  |  |  |  |  |  |  |  |  |  |  |  |  |  |  |  |  |  |  |  |
|  | HSCT |  |  |  |  |  |  |  |  |  |  |  |  |  |  |  |  |  |  |  |  |  |  |  |  |  |  |  |  |  |  |  |  |  |
| **Outcome** | Asymptomatic |  |  |  |  |  |  |  |  |  |  |  |  |  |  |  |  |  |  |  |  |  |  |  |  |  |  |  |  |  |  |  |  |  |
|  | Symptomatic survival |  |  |  |  |  |  |  |  |  |  |  |  |  |  |  |  |  |  |  |  |  |  |  |  |  |  |  |  |  |  |  |  |  |
|  | No serious infections |  |  |  |  |  |  |  |  |  |  |  |  |  |  |  |  |  |  |  |  |  |  |  |  |  |  |  |  |  |  |  |  |  |
|  | Died |  |  |  |  |  |  |  |  |  |  |  |  |  |  |  |  |  |  |  |  |  |  |  |  |  |  |  |  |  |  |  |  |  |
|  | Chromothrinsis |  |  |  |  |  |  |  |  |  |  |  |  |  |  |  |  |  |  |  |  |  |  |  |  |  |  |  |  |  |  |  |  |  |

RRI: Recurrent respiratory infections; HSV: Herpes simplex virus; VZV: Varicella-zoster virus; PD: Periodontal disease; AU: Aphthous ulcers; ITP: Immune thrombocytopenia purpura; CPAHA: Coombs-positive autoimmune hemolytic anemia; TD1: Type 1 diabetes; VI: Vaginal infections; UTI: Urinary tract infections; VSD: Ventricular septal defect; FT: Tetralogy of Fallot; CH: Congenital hypothyroidism; CBCC: Cutaneous basal cell carcinomas; ISCC: Invasive squamous cell carcinoma

**References**

1. Takaya J, Fujii Y, Higashino H, Taniuchi S, Nakamura M, and Kaneko K. A case of WHIM syndrome associated with diabetes and hypothyroidism. *Pediatr Diabetes*. (2009) 10(7):484-486. doi: 10.1111/j.1399-5448.2009.00503.x

2. Taniuchi S, Yamamoto A, Fujiwara T, Hasui M, Tsuji S, and Kobayashi Y. Dizygotic twin sisters with myelokathexis: mechanism of its neutropenia. *American journal of hematology*. (1999) 62(2):106-111. doi: 10.1002/(sici)1096-8652(199910)62:2<106::aid-ajh8>3.0.co;2-d

3. Hagan JB, and Nguyen PL. WHIM syndrome. *Mayo Clinic proceedings*. (2007) 82(9):1031. doi: 10.4065/82.9.1031

4. Badolato R, Dotta L, Tassone L, Amendola G, Porta F, Locatelli F, et al. Tetralogy of fallot is an uncommon manifestation of warts, hypogammaglobulinemia, infections, and myelokathexis syndrome. *The Journal of pediatrics*. (2012) 161(4):763-765. doi: 10.1016/j.jpeds.2012.05.058

5. Kawahara Y, Oh Y, Kato T, Zaha K, and Morimoto A. Transient Marked Increase of γδ T Cells in WHIM Syndrome After Successful HSCT. *Journal of clinical immunology*. (2018) 38(5):553-555. doi: 10.1007/s10875-018-0529-4

6. McDermott DH, Pastrana DV, Calvo KR, Pittaluga S, Velez D, Cho E, et al. Plerixafor for the Treatment of WHIM Syndrome. *The New England journal of medicine*. (2019) 380(2):163-170. doi: 10.1056/NEJMoa1808575

7. Saettini F, Notarangelo LD, Biondi A, and Bonanomi S. Neutropenia, hypogammaglobulinemia, and pneumonia: A case of WHIM syndrome. *Pediatr Int*. (2018) 60(3):318-319. doi: 10.1111/ped.13488

8. Aghamohammadi A, Abolhassani H, Puchalka J, Greif-Kohistani N, Zoghi S, Klein C, et al. Preference of Genetic Diagnosis of CXCR4 Mutation Compared with Clinical Diagnosis of WHIM Syndrome. *Journal of clinical immunology*. (2017) 37(3):282-286. doi: 10.1007/s10875-017-0387-5

9. Yoshii Y, Kato T, Ono K, Takahashi E, Fujimoto N, Kobayashi S, et al. Primary cutaneous follicle center lymphoma in a patient with WHIM syndrome. *Journal of the European Academy of Dermatology and Venereology : JEADV*. (2016) 30(3):529-530. doi: 10.1111/jdv.12927

10. Handisurya A, Schellenbacher C, Reininger B, Koszik F, Vyhnanek P, Heitger A, et al. A quadrivalent HPV vaccine induces humoral and cellular immune responses in WHIM immunodeficiency syndrome. *Vaccine*. (2010) 28(30):4837-4841. doi: 10.1016/j.vaccine.2010.04.057

11. Tassone L, Notarangelo LD, Bonomi V, Savoldi G, Sensi A, Soresina A, et al. Clinical and genetic diagnosis of warts, hypogammaglobulinemia, infections, and myelokathexis syndrome in 10 patients. *J Allergy Clin Immunol*. (2009) 123(5):1170-1173, 1173.e1171-1173. doi: 10.1016/j.jaci.2008.12.1133

12. Aprikyan AA, Liles WC, Park JR, Jonas M, Chi EY, and Dale DC. Myelokathexis, a congenital disorder of severe neutropenia characterized by accelerated apoptosis and defective expression of bcl-x in neutrophil precursors. *Blood*. (2000) 95(1):320-327. doi:

13. Siedlar M, Rudzki Z, Strach M, Trzyna E, Pituch-Noworolska A, Błaut-Szlósarczyk A, et al. Familial occurrence of warts, hypogammaglobulinemia, infections, and myelokathexis (WHIM) syndrome. *Arch Immunol Ther Exp (Warsz)*. (2008) 56(6):419-425. doi: 10.1007/s00005-008-0046-x

14. Tarzi MD, Jenner M, Hattotuwa K, Faruqi AZ, Diaz GA, and Longhurst HJ. Sporadic case of warts, hypogammaglobulinemia, immunodeficiency, and myelokathexis syndrome. *J Allergy Clin Immunol*. (2005) 116(5):1101-1105. doi: 10.1016/j.jaci.2005.08.040

15. Krill CE, Jr., Smith HD, and Mauer AM. CHRONIC IDIOPATHIC GRANULOCYTOPENIA. *The New England journal of medicine*. (1964) 270:973-979. doi: 10.1056/nejm196405072701902

16. McDermott DH, Gao JL, Liu Q, Siwicki M, Martens C, Jacobs P, et al. Chromothriptic cure of WHIM syndrome. *Cell*. (2015) 160(4):686-699. doi: 10.1016/j.cell.2015.01.014

17. Wetzler M, Talpaz M, Kellagher MJ, Gutterman JU, and Kurzrock R. Myelokathexis: normalization of neutrophil counts and morphology by GM-CSF. *Jama*. (1992) 267(16):2179-2180. doi:

18. Wetzler M, Talpaz M, Kleinerman ES, King A, Huh YO, Gutterman JU, et al. A new familial immunodeficiency disorder characterized by severe neutropenia, a defective marrow release mechanism, and hypogammaglobulinemia. *The American journal of medicine*. (1990) 89(5):663-672. doi: 10.1016/0002-9343(90)90187-i

19. Chae KM, Ertle JO, and Tharp MD. B-cell lymphoma in a patient with WHIM syndrome. *Journal of the American Academy of Dermatology*. (2001) 44(1):124-128. doi: 10.1067/mjd.2001.111337

20. Cipriani NA, Blair E, and Taxy JB. WHIM syndrome and oral squamous cell carcinoma. *Oral surgery, oral medicine, oral pathology, oral radiology, and endodontics*. (2010) 109(1):105-108. doi: 10.1016/j.tripleo.2009.08.011
